# Clinical and aesthetic outcomes of enamel matrix derivative and subepithelial connective tissue graft using the modified coronally advanced tunnel technique: a randomized controlled trial

**DOI:** 10.1590/1678-7765-2025-0775

**Published:** 2026-03-23

**Authors:** Fatma Altiparmak, Fatma Ucan Yarkac

**Affiliations:** 1 Karamanoglu Mehmetbey University Faculty of Dentistry Department of Periodontology Karaman Turkey Karamanoglu Mehmetbey University, Faculty of Dentistry, Department of Periodontology, Karaman, Turkey.; 2 Necmettin Erbakan University Faculty of Dentistry Department of Periodontology Konya Turkey Necmettin Erbakan University, Faculty of Dentistry, Department of Periodontology, Konya, Turkey.

**Keywords:** Gingival recession, Graft, Enamel matrix proteins, Minimally invasive surgical procedures, Subepithelial connective tissue

## Abstract

**Objectives:**

This randomized controlled clinical trial evaluated the short-term clinical outcomes of the modified coronally advanced tunnel technique combined with three different regenerative approaches—subepithelial connective tissue graft (as the gold standard), enamel matrix derivative (as a donor-site-sparing alternative), and their combination (to explore potential synergistic effects)—in treating recession type 1 gingival defects.

**Methodology:**

A total of 52 systemically healthy patients (aged 18–60) with 90 recession type 1 gingival defects were enrolled and randomly assigned to one of the three treatment groups: Group 1 (n=18; subepithelial connective tissue graft), Group 2 (n=19; enamel matrix derivative), and Group 3 (n=15; both materials). Primary outcome consisted of the percentage of root coverage at 6 months. Secondary outcomes included plaque index, gingival index, gingival recession depth, gingival recession width, clinical attachment level, keratinized tissue width, gingival thickness, wound healing index, probing depth, and root coverage aesthetic score. Clinical parameters were measured at baseline, 3, and 6 months, and aesthetic outcomes were assessed at 6 months.

**Results:**

All groups showed significant improvements in clinical parameters. Mean root coverage was 80.4% for Group 1, 76.2% for Group 2, and 73.6% for Group 3, with no significant differences among them. Keratinized tissue width significantly increased in Group 1 and Group 3, but not in Group 2. Mean root coverage aesthetic score values were 7.74 ± 2.35, 7.54 ± 2.39, and 7.62 ± 1.97, respectively (p > 0.05).

**Conclusion:**

Within the limitations of this study, including its short-term follow-up period and baseline differences in recession width, all three groups exhibited comparable clinical and aesthetic outcomes at 6 months. Clinical Trial Registration Number: NCT06504329

## Graphical abstract

**Figure:**
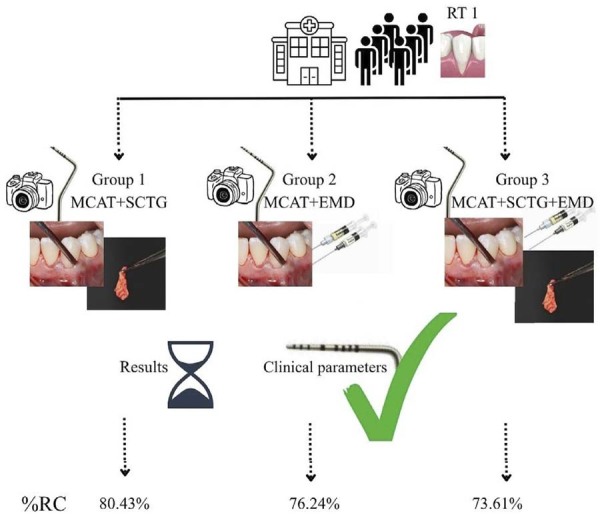

## Introduction

Gingival recession constitutes the apical displacement of the gingival margin beyond the cementoenamel junction (CEJ), resulting in root surface exposure.^1^ Highly prevalent, this condition affects a considerable proportion of the population irrespective of age, gender, or ethnicity.^2^ Recent epidemiological studies indicate that the prevalence of gingival recession in the midfacial region is remarkably high, reaching up to 99.7% at the patient level.^3^ While often perceived as a primary aesthetic issue, gingival recession may also lead to dentin hypersensitivity, root caries, and plaque accumulation. Moreover, it can be associated with a reduction in keratinized tissue width (KTW) which further compromises periodontal health.^1^ Notably, untreated gingival recession may progressively lead to further periodontal attachment loss over time, even under good oral hygiene.^4,5^

Due to these clinical implications, a variety of surgical techniques have been developed to achieve root coverage, including pedicle and soft tissue grafts.^6^ But attaining both predictable root coverage and optimal aesthetic outcomes remains a challenge, particularly for patients with high aesthetic expectations.^7^ In recent years, refinements in minimally invasive tunnel-based approaches have been proposed to enhance surgical flexibility and patient outcomes. Among these, the Mixed-Thickness Tunnel Access (MiTT) technique—performed via a linear vertical mucosal incision—has been introduced as a minimally invasive option applicable to both anterior and posterior regions.^8^ Within this evolving surgical context, the Modified Coronally Advanced Tunnel (MCAT) technique has emerged as a predictable and minimally invasive root-coverage approach. By preserving soft tissue integrity and vascularization, and enabling stable coronal advancement without vertical releasing incisions, MCAT has been associated with consistently favorable clinical outcomes. In contemporary periodontal plastic surgery, MCAT is most commonly combined with subepithelial connective tissue grafting (SCTG) to enhance soft-tissue thickness, flap stability, and long-term predictability.^9-11^

Several studies have shown that the combined MCAT + SCTG technique results in predictable coverage of adjacent gingival recessions, with a mean root coverage of 90–96% for Recession Type (RT) 1 defects and approximately 83% for RT2.^9,11-13^ Moreover, recent systematic reviews and meta-analyses have supported the overall predictability of tunnel-based root coverage approaches and have assessed whether adjunctive biologics (including enamel matrix derivative – EMD) provide additional benefit, concluding that results may vary depending on the surgical protocol, outcome definition, and defect characteristics.^14,15^

Despite the strong clinical performance of MCAT+SCTG, SCTG-based procedures present inherent limitations, most notably the requirement for a secondary donor site which may increase patient morbidity and may not provide sufficient graft volume in cases involving multiple gingival recessions.^16^ Accordingly, biomaterial-based approaches such as EMD have been explored as donor-site–sparing alternatives or adjuncts to further optimize healing and clinical outcomes. Among these biomaterials, EMD has been reported to enhance treatment outcomes when integrated into periodontal plastic surgical procedures.^5,17,18^ Preclinical studies and clinical observations suggest that EMD, applied alone or in combination with SCTG, may promote periodontal wound healing and support the formation of periodontal tissues.^17-19^ Recent clinical evidence, including a split-mouth randomized controlled trial with molecular evaluation, has further indicated that adjunctive EMD use with SCTG can positively influence early wound healing by modulating inflammatory markers.^5^ Moreover, numerous studies have indicated that EMD application may result in accelerated wound healing and reduced inflammation compared with sites treated without EMD, suggesting its clinical significance in regulating early wound healing.^20, 21^

Despite these advancements, randomized controlled trials providing a direct three-arm comparison of SCTG, EMD, and their combination within a MCAT protocol remain limited. Thus, this study addresses this gap by evaluating whether EMD may serve as an effective standalone alternative to the gold-standard SCTG, or whether their combination provides an additive clinical benefit in treating RT1 gingival recessions. We posited that all three treatment modalities would result in comparable percentages of root coverage (%RC) at the 6-month follow-up.

## Methodology

### Study protocol and ethical approval

This study presents the outcomes of a 6-month follow-up randomized controlled trial, conducted in strict accordance with the CONSORT statement to ensure transparent and high-quality reporting of parallel-group randomized trials. Participants were selected from individuals referred to the Department of Periodontology at Necmettin Erbakan University between January and July 2024. The trial was registered at ClinicalTrials.gov (NCT06504329). The primary outcome measure was defined as the percentage of root coverage (%RC) at the 6-month follow-up.

The study was conducted in accordance with the Declaration of Helsinki, as revised most recently in 2024, and was performed at a single academic institution. In accordance with national regulations, two ethical approvals were obtained: the first was from the Clinical Research Ethics Committee of Necmettin Erbakan University, Meram Faculty of Medicine (Approval No: 2023/1066, April 12, 2023), and the second was from the Turkish Medicines and Medical Devices Agency (Approval No: E-66175679-514.13.02-1163228, July 17, 2023). The use of subepithelial connective tissue grafts was authorized by the General Directorate of Health Services, Ministry of Health, Turkey (Approval No: E-56733164-202.99-236451757, January 5, 2024). Particularly for research involving human biological materials, the thorough approvals guaranteed compliance with regulatory requirements.

### Sample size calculation

Sample size was estimated prior to study initiation, with calculations based on the primary endpoint (%RC at 6 months). A power analysis for a three-arm, one-way ANOVA design was performed using G*Power software, referencing effect sizes reported in comparable clinical studies (f=0.40).^22-24^ Results indicated that a sample of 66 participants (22 per group) would achieve 80% power at an alpha level of 0.05, assuming a large effect size (f=0.40).

Following study completion, a post-hoc power analysis was conducted based on the achieved sample size. Assuming a large Cohen’s effect size (f=0.40), the actual power was calculated as 0.92, confirming that the study was supported by robust statistical power.

### Participant selection and randomization

Participants were enrolled according to the following inclusion criteria: age between 18 and 60 years, good systemic and periodontal health, non-smoking status, and presence of a clearly detectable CEJ at the selected sites; no history of previous surgical interventions in the study sites; absence of periodontal pockets >3 mm and occlusal trauma; presence of single or multiple RT1 gingival recessions, classified according to Cairo, et al.^13^ (2011), located at anterior and posterior teeth in both the maxilla and/or mandible, with aesthetic concerns or dentinal hypersensitivity.

Exclusion criteria comprised pregnancy or lactation, the use of antibiotics within the prior six months, and systemic conditions that may contraindicate surgical interventions. Additionally, sites with non-carious cervical lesions or non-detectable CEJs were excluded, as were participants who failed to meet the inclusion criteria or chose not to participate.

After obtaining written informed consent, eligible participants were randomly assigned to one of three treatment groups using a computer-generated randomization sequence. Allocation concealment was ensured by an independent researcher (F.U.Y.) using sequentially numbered, opaque, sealed envelopes (SNOSE). The envelope was opened only immediately after the tunnel preparation was completed and just before application of the assigned material(s), thereby preventing bias during baseline clinical measurements and the initial surgical steps. The researcher responsible for allocation concealment (F.U.Y.) had no role in the surgical procedures, clinical measurements, or outcome assessment. Due to the nature of the interventions (SCTG harvesting and/or EMD application), blinding of the surgeon and participants was not feasible. To minimize measurement bias, all clinical recordings were performed by a single calibrated investigator (F.A.) using a standardized protocol, and follow-up assessments at 3 and 6 months were recorded without reference to previous baseline values or the allocation list.

### Examiner calibration and reliability

All surgical procedures and clinical measurements were performed by a single experienced periodontist (F.A.) to ensure methodological consistency and eliminate inter-examiner variability. Intraexaminer reliability was assessed on 10 volunteer participants (not included in the study) by repeating the measurements in two sessions, conducted 24 hours apart. Intraexaminer reliability was evaluated using intraclass correlation coefficients (ICC), demonstrating high reproducibility for KTW (ICC=0.92) and PD (ICC=0.93).

### Clinical evaluations

All participants underwent nonsurgical periodontal therapy before enrollment in the surgical phase, including detailed oral hygiene instructions and supragingival scaling, to achieve and maintain optimal plaque control. Surgical interventions were initiated only after confirming satisfactory oral hygiene and adequate supragingival plaque control. Clinical measurements were performed at baseline and at the 3- and 6-month follow-up visits using a Williams periodontal probe (Hu-Friedy, Chicago, IL, USA).

- Primary outcome measure

Primary outcome was the %RC at 6 months. %RC was calculated using the standardized formula.^25^


$$\%\mathrm{RC}=\frac{\text{ pre operative GRD - post operative GRD }}{\text{ pre operative GRD }}\times 100$$

- Secondary Outcome Measures

Secondary outcome measures included clinical, aesthetic, and healing-related parameters.

Full-mouth Plaque Index (PI) and Gingival Index (GI) were recorded at three sites per tooth (mesial, distal, and mid-buccal) according to Silness and Löe, with third molars excluded from the analysis.^26^ Gingival recession width (GRW) was measured horizontally at the widest point of the recession, while gingival recession depth (GRD) was measured from the cementoenamel junction (CEJ) to the gingival margin. Keratinized tissue width (KTW) was assessed as the distance from the gingival margin to the mucogingival junction. Probing depth (PD) was measured from the gingival margin to the apical extent of the sulcus, and clinical attachment level (CAL) was measured from the CEJ to the apical extent of the sulcus.

Gingival thickness (GT) was measured at the mid-buccal aspect, 2 mm apical to the gingival margin, using a canal instrument with a rubber stopper. The distance between the instrument tip and the stopper was measured with a digital caliper and rounded to the nearest 0.1 mm.^27^

Wound healing was evaluated two weeks after surgery following suture removal using the Wound Healing Index according to Huang, et al.^28^ (2005). Healing was classified as Score 1 (uneventful healing without edema, erythema, suppuration, patient discomfort, or flap dehiscence), Score 2 (uneventful healing with slight edema, erythema, patient discomfort, or flap dehiscence, without suppuration or infection), or Score 3 (poor healing characterized by pronounced edema, erythema, patient discomfort, flap dehiscence, or infection).

Aesthetic outcomes were assessed at the 6-month follow-up using the Root Coverage Aesthetic Score (RES), a composite index evaluating soft tissue appearance following root coverage procedures.^29^ RES encompasses five parameters: level of the gingival margin (0, 3, or 6 points), marginal tissue contour (0 or 1), soft tissue texture (0 or 1), alignment of the mucogingival junction (0 or 1), and gingival color integration (0 or 1), with higher total scores indicating superior aesthetic outcomes.

### Surgical treatment

All surgical procedures were performed under 3.5× magnification using microsurgical instruments (Hu-Friedy, USA) and magnification loupes (Carl Zeiss Meditec AG, Germany). Local anesthesia was achieved with articaine (40 mg/mL) containing epinephrine (0.01819 mg/mL) (Ultraver D-S Fort, Haver, Istanbul, Turkey). MCAT was performed as described by Sculean, et al.^30^ (2014).

Intrasulcular incisions were made with microsurgical scalpels, followed by elevation of a full-thickness mucoperiosteal flap beyond the mucogingival junction while preserving the interdental papillae. To facilitate tension-free coronal flap advancement, connective tissue attachments and muscle insertions were meticulously released. For adjacent multiple recession defects, the flap was laterally extended and tunneled beneath papillae, ensuring papillary integrity and avoiding perforation.

**Figure 1:**
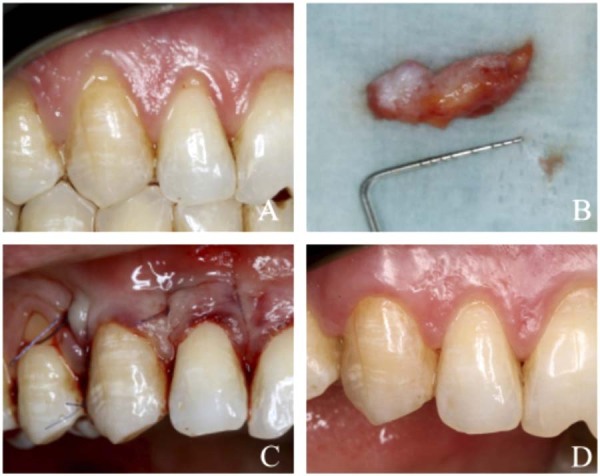
Clinical case presentation (MCAT + SCTG) (A) Gingival recession defects on teeth 12 and 13 at baseline. (B) Subepithelial connective tissue graft (SCTG) harvested from the palate. (C) Final view immediately following the surgical procedure. (D) Final aaesthetic and clinical outcome at the 6-month postoperative follow-up.

**Figure 2:**
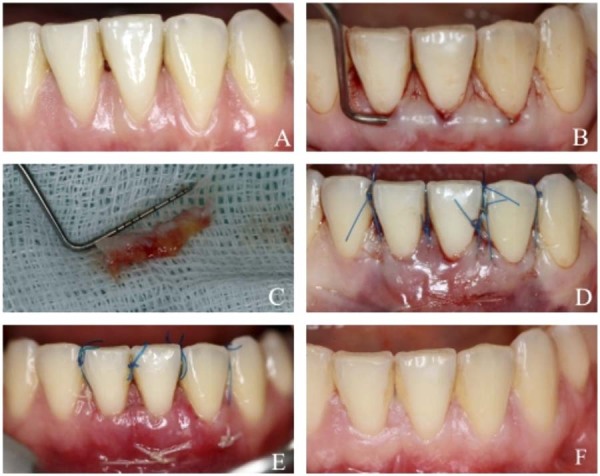
Clinical case presentation (MCAT + SCTG + EMD) (A) Gingival recession defects on teeth 41, 31, and 32 at baseline. (B) Preparation of the tunnel for graft placement. (C) Subepithelial connective tissue graft (SCTG) harvested from the palate and storage in enamel matrix derivative (EMD). (D) Final view immediately following the surgical procedure. (E) Healing at the 2-week postoperative follow-up. (F) Final aesthetic and clinical outcome at the 6-month postoperative follow-up.

**Figure 3:**
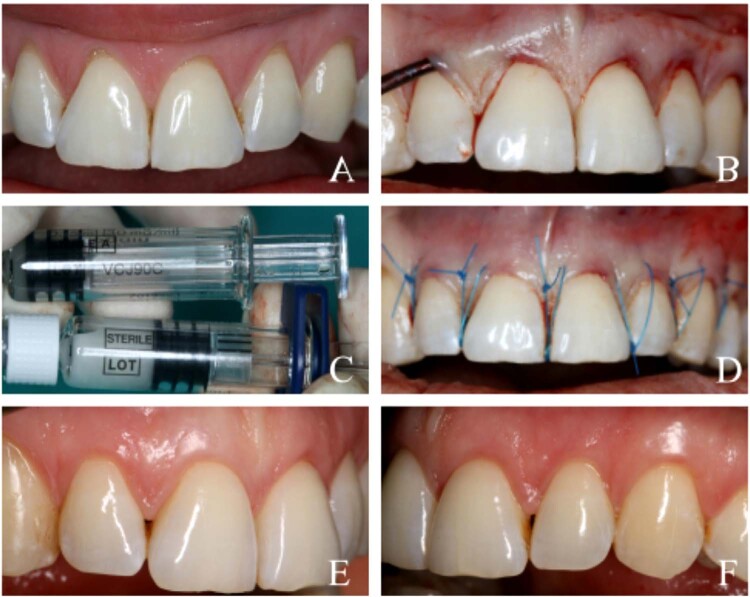
Clinical case presentation (MCAT + EMD) (A) Gingival recession defects on teeth 12, 11, 21, 22, and 23 at baseline. (B) Preparation of the tunnel. (C) Enamel matrix derivative (EMD). (D) Final view immediately following the surgical procedure. (E-F) Final aesthetic and clinical outcome at the 6-month postoperative follow-up.

For Groups 1 (MCAT + SCTG) (Figure 1) and 3 (MCAT + SCTG + EMD) (Figure 2), a 1–1.5 mm thick SCTG was harvested from the palatal region using single-incision.^16^ In Group 1, the graft was stored in sterile saline until placement. In Group 3, following harvesting, the SCTG was immersed in EMD gel for 5 minutes prior to placement, allowing biological conditioning of the graft.^17^ Palatal hemostasis was achieved by gauze compression, followed by closure with 4-0 resorbable sutures (Boz Cerrahi Sütür, Istanbul, Turkey).

For Groups 2 (MCAT + EMD) (Figure 3) and 3, EMD (Emdogain^®^, Straumann) was applied in accordance with manufacturer’s instructions. Following root surface decontamination, 24% EDTA gel (PrefGel^®^, Straumann) was applied to the exposed root surfaces for 2 minutes to remove the smear layer. The root surfaces were then thoroughly rinsed with sterile saline and gently dried using sterile gauze to obtain a moisture-controlled, blood-free environment. EMD was subsequently applied with a syringe to the conditioned root surfaces and the underside of the tunnel flap to achieve complete and uniform coverage of all exposed root surfaces from the most apical point to the cemento-enamel junction. In cases involving multiple adjacent recession defects, moisture control was achieved sequentially using sterile gauze and high-volume suction, and EMD application was performed in a stepwise manner to maintain a blood-free field. EMD was left in situ and not rinsed off prior to flap advancement and suturing.

SCTG (in Groups 1 and 3) was positioned within the tunnel and stabilized with guiding sutures. The tunnel flap was then coronally advanced and secured with 6-0 nylon sling sutures (Boz Cerrahi Sutur, Istanbul, Turkey), ensuring complete coverage of the graft and the recession defect, when applicable**.** Following suturing, gentle pressure with moist gauze was applied for 5 minutes to minimize dead space and prevent excessive blood clot formation.

### Postoperative care and controls

Postoperative care included oral analgesics (Flurbiprofen 100 mg, Turkey, twice daily for 3 days), oral antibiotics (Augmentin 1000 mg tb. GlaxoSmithKline, Turkey, twice daily for 7 days), and 0.12% chlorhexidine gluconate mouthwash (Kloroben Drogsan, Turkey), prescribed twice daily for 1 minute over 21 days.

Patients were instructed to avoid vigorous rinsing, manipulation of the surgical site, mastication in the treated area, and consumption of hard or acidic foods until suture removal. Suture removal was performed between 7–14 days post-op for palatal donor sites and on day 14 for recipient sites. Mechanical plaque control was reintroduced on day 3 post-surgery using an ultra-soft toothbrush and the roll technique, while routine oral hygiene practices were gradually reinstated after one month. Oral hygiene reinforcement was provided at each follow-up visit, and professional supragingival prophylaxis was performed when necessary to maintain optimal plaque control.

### Statistical analysis

Data were analyzed using IBM SPSS Statistics v27 (IBM Corp., Armonk, NY, USA). Continuous variables were presented as mean, standard deviation, median, minimum, and maximum values, while categorical variables were expressed as frequency (n) and percentage (%). Normality of continuous variables was assessed by Shapiro-Wilk testing. Intergroup comparisons used Kruskal-Wallis H test followed by Dunn’s post-hoc test for significant results. Intragroup comparisons were performed using the Friedman and Wilcoxon tests. Categorical variables were analyzed using Pearson’s chi-squared test. Impact of age, GT, localization, and KTW on %RC was assessed by linear regression analysis. Given that multiple recession sites were included per patient, an additional sensitivity analysis was performed using a linear mixed-effects model with a random intercept for patient. Treatment group was entered as a fixed factor, and baseline GRW was included as a covariate. Statistical significance was set at p<0.05.

## Results

**Figure 4:**
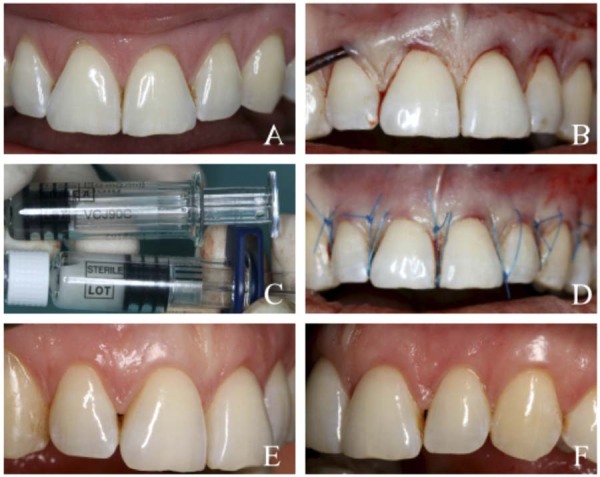
Flow chart.

During the study period, 14 participants were excluded due to failure to attend scheduled follow-up visits. Consequently, 52 participants with a total of 90 gingival recession defects completed the 6-month follow-up and were included in the final analysis—Group 1: 18 participants (35 defects), Group 2: 19 participants (31 defects), and Group 3: 15 participants (24 defects). Uneven patient distribution and defects across the groups at the final assessment resulted from the patient-based randomization protocol and the differing number of recession defects per participant at baseline. Our final cohort included 24 males and 28 females participants aged 18–60 years (Figure 4). Analyses were performed at both patient- and defect-levels to increase the validity and precision of the clinical outcomes.

### Descriptive data of the participants

A total of 52 systemically healthy participants (24 males, 28 females; mean age: 41.14±9.72 years) presenting 90 RT1 gingival recession defects were included in the final analysis. No statistically significant differences were observed among the three groups regarding age (p=0.732) or gender (p=0.95). Distribution of defects according to tooth type (anterior, premolar, molar) and tooth side (right/left) was comparable across groups (p>0.05). However, arch distribution showed a statistically significant difference, with Group 3 presenting a lower proportion of maxillary defects (p=0.020). Additionally, flap design differed significantly among groups, with Group 1 exhibiting a higher proportion of multiple recession cases (p=0.045) (Table 1).

**Table 1:** Baseline characteristics and recession site distribution of study groups.

Characteristics	Group 1	Group 2	Group 3	p
**Age** (years)	42.03±5.71	41.94±9.41	39.29±12.7	0.732
**Sex** (male/female)	12/6	7/12	5/10	0.95
**Teeth**, n (%)				
Anterior	16 (37.2)	15( 34.9)	12 (27.9)	0.388
Premolar	19 (42.2)	14 (31.1)	12 (26.7)	
Molar	0	2 (100)	0	
**Arch**, n (%)				
Maxilla	20 (40)	22 (44)	8(16)	0.020*
Mandible	15 (37.5)	9 (22.5)	16(40)	
**Side**, n (%)				
Right	23 (44.2)	15 (28.8)	14 (26.9)	0.363
Left	12 (31.6)	16 (42.1)	10 (26.3)	
**Flap desig**n, n (%)				
Single	4 (17.4)	10 (43.5)	9 (39.1)	0.045*
Multiple	31 (46.3)	21 (31.3)	15 (22.4)	

Notes: *Bold values indicate statistical significance (p < 0.05, Chi-squared test).

Abbreviations: Group 1: Modified coronally advanced tunnel (MCAT) + Subepithelial connective tissue graft (SCTG) Group 2: MCAT + Enamel matrix derivative (EMD) Group 3: MCAT + SCTG + EMD n: number of recession sites.

### Clinical and wound healing outcomes

All treatment groups showed significant improvements in most clinical parameters from baseline to the 6-month follow-up. PD significantly decreased while GRD and GRW showed significant reductions. Similarly, CAL improved significantly in all groups. KTW also increased over time in all groups; however, the change in Group 2 was not statistically significant. The primary outcome of %RC at 6 months showed no statistically significant difference between groups (p=0.701). Mean %RC at 6 months was 80.43±26.89% (95% CI: 71.21%–89.65%) for Group 1, 76.24±32.32% (95% CI: 64.38%–88.10%) for Group 2, and 73.61±30.22% (95% CI: 60.80%–86.42%) for Group 3.

In the GRW-adjusted mixed model, baseline GRW was significantly associated with 6-month %RC (β=−8.04 per 1-mm increase; p=0.006); however, adjusted intergroup comparisons remained non-significant (SCTG vs EMD: p=0.266; SCTG vs SCTG + EMD: p=0.287).

No adverse events or clinical complications such as severe edema, infection, flap dehiscence, or allergic reactions, were observed in any of the patients during the 6-month follow-up period. Postoperative healing was uneventful for all participants in all three treatment groups.

Intergroup comparisons found no notable differences in GI and PI at any time point; however, statistically significant intergroup differences were detected for GT at both baseline and 6 months. At baseline, GT was significantly higher in Group 1 compared with Group 3 (p<0.05), but by the 6-month follow-up this difference was no longer statistically significant (p>0.05), with both SCTG-containing groups (Group 1 and Group 3) exhibiting significantly higher GT than Group 2 (p<0.05). Intergroup comparisons at the 14-day follow-up revealed a statistically significant reduction in wound healing index scores, with Group 2 showing the best scores, followed by Group 1 and Group 3. Table 2 summarizes the detailed parameters for all groups.

**Table 2:** Clinical, aesthetic, and wound healing parameters.

Parameters	Timepoint	MCAT+SCTG (n=35)	MCAT+EMD (n=31)	MCAT+SCTG+EMD (n=24)	p
Probing Depth (mm)	Baseline ( t_1_)	2.14^a^ ±0.55	1.76^b^±0.5	1.63^ab^±0.49	**<0.001**^**a vs. b,a vs. ab**^
	6^th^ Month (t_3_)	1.8±0.58	1.32±0.48	1.23±0.47	**<0.001**^**a vs. b, a vs. ab**^
**p value t**_**1**_ **vs. t**_**3**_		**<0.018**	**<0.002**	**<0.001**	
Gingival Recession Depth (mm)	Baseline (t_1_)	2.14±0.64	1.82±0.74	2.15±0.68	NS
	3^rd^ Month (t_2_)	0.67±0.8	0.66±0.83	0.85±0.8	NS
	6^th^ Month (t_3_)	0.54±0.74	0.61±0.83	0.67±0.76	NS
**p value t**_**1**_ **vs. t**_**3**_		**<0.001**	**<0.001**	**<0.001**	
Gingival Recession Width (mm)	Baseline (t_1_)	3.43^a^±0.96	2.97^b^±1.23	2.86^ab^±1.19	**0.04**^**a vs. b, a vs ab**^
	3^rd^ Month (t_2_)	1.36±1.55	1.52±1.82	1.77±1.71	NS
	6^th^ Month (t_3_)	1.24±1.57	1.45±1.84	1.6±1.8	NS
**p value t**_**1**_ **vs. t**_**3**_		**<0.001**	**<0.001**	**<0.001**	
Clinical Attachment Level (mm)	Baseline (t_1_)	3.81^a^±1.04	3.34^b^±0.91	3.27^ab^±0.72	**0.013**^**a vs. b, a vs. ab**^
	6^th^ Month (t_3_)	2.31±1.06	1.97±0.94	1.83±0.82	NS
**p value t**_**1**_ **vs. t**_**3**_		**<0.001**	**<0.001**	**<0.001**	
Keratinized Tissue Width (mm)	Baseline (t_1_)	3.34±1.63	3.76±1.36	3.85±2.21	NS
	3^rd^ Month (t_2_)	3.99±1.81	3.89±1.56	4.63±2.24	NS
	6^th^ Month (t_3_)	4.46±1.66	4.05±1.29	5.00±2.17	NS
**p value t**_**1**_ **vs. t**_**3**_		**<0.001**	**0.511**	**<0.001**	
Gingival Thickness (mm)	Baseline (t_1_)	0.93^a^±0.28	0.8^b^±0.24	0.78^ab^±0.41	**0.017**^**a vs. ab**^
	6^th^ Month (t_3_)	1.59^a^±0.32	1.18^b^±0.29	1.45^ab^±0.43	**0.001**^**a vs. b, b vs. ab**^
**p value t**_**1**_ **vs. t**_**3**_		**<0.001**	**<0.001**	**<0.001**	-
Root Coverage (%)	6^th^ Month	80.43%	76.24%	73.61%	0.701
	SD	±26.89	±32.32	±30.22	
Root Coverage Aesthetic Score (total)	6^th^ Month	7.74±2.35	7.54±2.39	7.62±1.97	0.930
Wound Healing Index	14-Day	1.31±0.47	1.10±0.30	1.58±0.50	**<0.001**

Intergroup comparisons used Kruskal-Wallis H testfollowed by Dunn’s post-hoc test for significant results. Intragroup comparisons used Friedman and Wilcoxon tests. Categorical variables were analyzed using Pearson’s chi-squared test. Statistical significance was set at p < 0.05. Continuous variables are presented as mean ± standard deviation (SD).

Abbreviations: MCAT, modified coronally advanced tunnel; SCTG, subepithelial connective tissue graft; EMD, enamel matrix derivative; t1, baseline ; t2,3rd month; t3, 6th month; NS: no significant; mm, millimeter; n, recession site; SD, standard deviation.

### Root coverage aesthetic score (RES)

Mean RES values at the 6-month follow-up were 7.74 2.35 in Group 1, 7.54±2.39 in Group 2, and 7.62±1.97 in Group 3, with no significant intergroup differences (p=0.930) (Table 2).

### Intergroup comparison of changes

Table 3 presents the changes in clinical parameters from baseline to 6 months across the different groups. No significant differences were found between the groups regarding changes in GRD, PD, or CAL. However, a significant difference was observed in GRW reduction, with Group 1 showing a greater decrease compared with the other groups (p<0.05). Changes in KTW also differed significantly between groups, with Group 2 showing less improvement than Group 1 and Group 3 (p<0.05).

**Table 3:** Intergroup comparison of changes from baseline to 6 months.

		MCAT+SCTG (n=35)	MCAT+EMD (n=31)	MCAT+SCTG+EMD (n=24)	p
Gingival Recession Depth	Mean±SD	1.60±0.86	1.21±0.63	1.48±0.77	0.100
Baseline - 6^th^ Month	Median	2.0 (0–3)	1.0 (0–3)	1.25 (0–3)	
	(Min–Max)				
Gingival Recession Width	Mean±SD	2.19±1.49	1.52±1.13	1.26±1.42	**0.036**
Baseline - 6^th^ Month	Median	2.0 (0–5)	2.0 (0–3)	0.5 (0–4)	
	(Min–Max)				
Probing Depth	Mean±SD	0.34±0.76	0.44±0.67	0.40±0.53	0.814
Baseline - 6^th^ Month	Median	0.0 (–1–2)	1.0 (–1–1)	0.0 (–0.5–1)	
	(Min–Max)				
Clinical Attachment Level	Mean±SD	1.49±1.04	1.37±0.80	1.44±0.86	0.717
Baseline - 6^th^ Month	Median	1.5 (–0.7–4)	1.0 (0–4)	1.25 (0–3)	
	(Min–Max)				
Keratinized Tissue Width	Mean±SD	1.11±0.87	0.29±0.83	1.15±0.45	**<0.001**
Baseline - 6^th^ Month	Median	1.0 (–0.5–4)	0.0 (–2–2)	1.0 (0–2)	
	(Min–Max)				

Abbreviations: MCAT, modified coronally advanced tunnel; SCTG, subepithelial connective tissue graft; EMD, enamel matrix derivative; SD, standard deviation; Min, minimum; Max, maximum; n, number of recession site. *Bold values indicate statistical significance (p < 0.05, Kruskal-Wallis H test).

### Regression analysis

Influence of baseline age, GT, tooth localization, and KTW on RC% was assessed using linear regression analysis (Table 4). Analysis indicated that both age and KTW significantly affected RC% (p<0.05). Specifically, for each unit increase in age, RC% experienced a reduction of 1.49 times (B=-1.49, 95% CI: -2.187 to -0.793; p<0.001), whereas a rise in KTW resulted in a 6.12-fold enhancement in RC% (B=6.127, 95% CI: 2.052 to 10.202; p=0.004). GT and localization were not significant predictors (p>0.05).

**Table 4:** Regression analysis of factors affecting root coverage percentage.

	B	S.E.	Beta	t	p	%95 confidence interval for B
Age	-1.49	0.351	-0.467	-4.251	**<0.001**	-2.187 -0.793
Gingival Thickness	-0.837	9.781	-0.009	-0.086	0.932	-20.284 -18.61
Location	-4.061	6.404	-0.069	-0.634	0.528	-16.794 -8.672
Keratinized Tissue Width	6.127	2.049	0.357	2.99	**<0.004**	2.052 -10.202

Abbreviations: B, regression coefficient; S.E, Standard error; Beta, standardized regression coefficient; t, test statistic.

## Discussion

This randomized controlled trial investigated the short-term clinical outcomes of MCAT combined with SCTG, EMD, and their combination in treating RT1 gingival recessions. Overall, the results confirm that MCAT is an effective and predictable minimally invasive approach, producing meaningful clinical improvements irrespective of the adjunctive biomaterial used. However, the absence of statistically significant intergroup differences in the primary outcome (%RC at 6 months) suggests that adjunctive EMD provided no measurable clinical advantage over SCTG alone. This finding is consistent with prior randomized controlled trials and evidence syntheses reporting limited additive clinical benefits of EMD when used in conjunction with MCAT and SCTG.^21,31-33^

While the clinical predictability of root coverage procedures has improved considerably, the biological nature of the soft tissue attachment formed on the root surface remains incompletely understood. Histological evidence suggests that various types of tissue attachment, including periodontal regeneration, may develop over recession defects following SCTG application with or without adjunctive EMD.^34,35^ In this context, the CAL gain observed across all treatment modalities is consistent with recent reports, such as Górski, et al. (2022), and supports the biological stability of the newly established attachment apparatus.^36^ Clinically, stable attachment formation is associated with reduced dentin hypersensitivity^37^ and contributes to a more favorable long-term prognosis for treated teeth.^13^ Moreover, the minimal changes in PD and the maintenance of shallow, healthy sulcus levels indicate that the applied surgical approaches did not adversely affect sulcus morphology. This outcome ensures a cleansable micro-environment, thereby facilitating effective plaque control and minimizing the risk of future inflammation-mediated attachment loss.^38^ Regarding keratinized tissue outcomes, available evidence remains inconclusive on the consistent adjunctive benefit of EMD. Górski, et al.^36^ (2022) reported no significant contribution of EMD to KTW gain in MCAT-based procedures, a finding corroborated by systematic reviews by Stähli, et al.^39^ (2020) and Mauricio, et al.^32^ (2021). Consistent with these observations, this study suggests that increases in KTW are primarily attributable to SCTG-related phenotype modification rather than to the adjunctive application of EMD.^40^

The decisive role of GT in marginal stability and wound healing has been well documented. GT is a critical determinant in root coverage procedures because it directly influences flap stability and the biological response during early healing. Thicker tissues exhibit increased resistance to inflammatory cell infiltration and mechanical challenges, whereas thin phenotypes are more susceptible to collagen degradation, marginal shrinkage, and recession relapse.^30^ Our findings show that using EMD alone resulted in limited GT augmentation compared with approaches incorporating SCTG. Although studies specifically addressing the assocation between GT and clinical outcomes in tunnel techniques remain scarce,^30,41^ current evidence consistently supports the role of SCTG as a powerful phenotype modifier.^42^ As highlighted by Zucchelli, et al.^42^ (2014) and further reinforced by the recent decision-making framework proposed by Aroca, et al.^30^ (2025), SCTG compensates for baseline tissue deficiencies, enhances flap stability, and contributes to the long-term preservation of marginal tissues. Moreover, the long-term clinical durability of SCTG has been highlighted in a recent 37-year retrospective evaluation, which showed that phenotype modification achieved through connective tissue grafting can maintain gingival margin stability over several decades.^43^ Despite baseline differences in GT—particularly between Group 1 and Group 3—, linear regression analysis revealed that initial GT was not a statistically significant predictor of 6-month %RC. This suggests that the standardized MCAT approach, when combined with appropriate biomaterials, may attenuate the clinical impact of baseline phenotypic variations and GT differences by enhancing wound stability during the early healing phase while still achieving clinically satisfactory short-term root coverage outcomes. These findings align with previous reports by Aroca, et al.^41^ (2021) and Stefanini, et al.^44^ (2018), who advocated the selective use of SCTG, specifically in sites exhibiting a thin phenotype to optimize healing dynamics, marginal stability, and aesthetic outcomes.

EMD has been shown to play a role in wound healing by promoting soft tissue regeneration and angiogenic activity.^17,21^ However, its biological effectiveness is critically dependent on stable adsorption to a clean, blood-free root surface.^17,36^ Several clinical studies have evaluated the effects of EMD on early wound healing, finding that its topical application may positively influence periodontal soft tissue healing in the early postoperative period.^5,17,20^ From a biological perspective, these effects have been primarily attributed to the modulation of angiogenic and inflammatory signaling pathways, including transient upregulation of growth factors such as VEGF and PDGF, rather than to a direct and consistent improvement in clinical healing outcomes.^5^ Nevertheless, clinical evidence remains inconsistent. In a randomized clinical trial evaluating the use of MCAT combined with SCTG with or without adjunctive EMD to treat single and multiple gingival recessions, Stähli, et al. reported similar trends in inflammatory biomarkers between groups and failed to sow a significant clinical or immunological advantage associated with EMD application.^39^ These findings suggest that molecular changes observed during early healing do not necessarily translate into measurable clinical benefits under minimally invasive surgical conditions. During MCAT preparation, limited access and micro-environmental conditions may increase the risk of blood contamination, potentially compromising EMD adsorption and biological performance. Here, the higher early wound healing scores observed in Group 3 may therefore be associated with increased procedural complexity, prolonged surgical duration, and difficulties in maintaining a blood-free surgical field, rather than with a direct biological effect of EMD itself. In line with this interpretation, our findings are consistent with previous clinical studies and long-term cohort data indicating that the adjunctive use of EMD in combination with MCAT and SCTG does not confer a significant additional benefit on early wound healing outcomes.^39,45^

A key limitation of this study concerns the statistically significant difference in baseline GRW between the study groups. Baseline recession dimensions have been recognized as potential determinants of root coverage success; therefore, this imbalance may represent a source of residual confounding in intergroup comparisons. Nevertheless, the primary outcome (%RC at 6 months) did not differ significantly between groups, and evidence from a randomized controlled clinical trial by Bakhishov, et al.^46^ (2021) indicates that baseline GRW did not exert a statistically significant effect on the percentage of root coverage achieved. Additionally, the overall pattern of outcomes observed was not suggestive of a systematic disadvantage for sites presenting with wider baseline recession defects, arguing against an explanation driven solely by initial defect size. Collectively, these observations support the overall consistency of our comparative conclusions and are compatible with the clinical ability of SCTG to partially offset anatomical challenges associated with wider recession defects via increased soft tissue volume and phenotype modification.^47^

Baseline distribution of single and multiple gingival recession defects between the study groups also requires consideration. In multiple-defect cases, the wider lateral extension of the tunnel flap may facilitate passive flap displacement and more stable coronal positioning, whereas limited extension in isolated defects may restrict flap mobility.^14^ Although the inclusion of both defect types represents a methodological limitation, the efficacy of MCAT for both isolated and multiple recessions has been well documented.^33,48-50^ Moreover, strict adherence to standardized surgical principles and the performance of all procedures by a single experienced clinician minimized operator-related variability. Absence of significant differences in the primary outcome (%RC at 6 months) further suggests a limited overall impact of these baseline variations on the comparative clinical results.

Several additional limitations should be acknowledged. First, the follow-up period was limited to 6 months; therefore, long-term stability and the potential for late marginal changes or relapse cannot be inferred. Second, baseline imbalances (including GRW and the distribution of single versus multiple defects) may have introduced residual confounding despite randomization. To mitigate this concern, a GRW-adjusted linear mixed-effects sensitivity analysis accounting for intra-subject clustering was performed. The conclusions for the primary outcome remained unchanged, supporting the robustness of the findings. Third, outcomes were assessed clinically, and histologic confirmation of the type of attachment and regeneration was not feasible. Finally, future studies with longer observation periods and standardized reporting of biologic and patient-centered endpoints are needed to clarify the biological mechanisms underpinning root coverage outcomes with and without adjunctive EMD.

## Conclusions

Within the limitations of this short-term study, the MCAT technique proved effective in treating RT1 gingival recessions, with consistent clinical and aesthetic improvements. Adjunctive use of EMD provided no additional benefits, whereas SCTG remained the primary determinant of keratinized tissue gain. Longer-term randomized controlled trials with larger cohorts are needed to validate these outcomes and further clarify the role of adjunctive biomaterials in root coverage procedures.

## References

[1] Marschner F, Lechte C, Kanzow P, Hraský V, Pfister W (2025). Systematic review and meta-analysis on prevalence and risk factors for gingival recession. J Dent.

[2] Barootchi S, Tavelli L (2022). Tunneled coronally advanced flap for the treatment of isolated gingival recessions with deficient papilla. Int J Esthet Dent.

[3] Barootchi S, Tavelli L, Vinueza MEG, Sabri H, Andrade C, Pinto N (2025). Autologous platelet concentrates in root coverage procedures. Periodontol 2000.

[4] Chambrone L, Tatakis DN (2016). Long-term outcomes of untreated buccal gingival recessions: a systematic review and meta-analysis. J Periodontol.

[5] Dias AT, Menezes CC, Kahn S, Fischer RG, Figueredo CM, Fernandes GV (2022). Gingival recession treatment with enamel matrix derivative associated with coronally advanced flap and subepithelial connective tissue graft: a split-mouth randomized controlled clinical trial with molecular evaluation. Clin Oral Investig.

[6] Pini Prato G, Di Gianfilippo R (2023). Challenges and success in periodontal plastic surgery. J Clin Periodontol.

[7] Pradhan S, Shetty N, Kamath D (2022). Comparison of coronally advanced flap with chorion membrane vs coronally advanced flap with connective tissue graft in the treatment of multiple gingival recessions: a split-mouth randomised controlled study. F1000Res.

[8] Marques T, Santos N, Sousa M, Fernandes JC, Fernandes GV (2023). Mixed-Thickness Tunnel Access (MiTT) through a linear vertical mucosal incision for a minimally invasive approach for root coverage procedures in anterior and posterior sites: technical description and case series with 1-year follow-up. Dent J (Basel).

[9] Aroca S, Molnár B, Windisch P, Gera I, Salvi GE, Nikolidakis D (2013). Treatment of multiple adjacent Miller class I and II gingival recessions with a modified coronally advanced tunnel (MCAT) technique and a collagen matrix or palatal connective tissue graft: a randomized, controlled clinical trial. J Clin Periodontol.

[10] Sculean A, Cosgarea R, Stähli A, Katsaros C, Arweiler NB, Brecx M (2014). The modified coronally advanced tunnel combined with an enamel matrix derivative and subepithelial connective tissue graft for the treatment of isolated mandibular Miller Class I and II gingival recessions: a report of 16 cases. Quintessence Int.

[11] Aroca S, Keglevich T, Nikolidakis D, Gera I, Nagy K, Azzi R (2010). Treatment of class III multiple gingival recessions: a randomized-clinical trial. J Clin Periodontol.

[12] Sculean A, Cosgarea R, Stähli A, Katsaros C, Arweiler NB, Miron RJ (2016). Treatment of multiple adjacent maxillary Miller Class I, II, and III gingival recessions with the modified coronally advanced tunnel, enamel matrix derivative, and subepithelial connective tissue graft: a report of 12 cases. Quintessence Int.

[13] Cairo F, Nieri M, Cincinelli S, Mervelt J, Pagliaro U (2011). The interproximal clinical attachment level to classify gingival recessions and predict root coverage outcomes: an explorative and reliability study. J Clin Periodontol.

[14] Tavelli L, Barootchi S, Nguyen TV, Tattan M, Ravidà A, Wang HL (2018). Efficacy of tunnel technique in the treatment of localized and multiple gingival recessions: a systematic review and meta-analysis. J Periodontol.

[15] Cairo F, Barootchi S, Tavelli L, Barbato L, Wang HL, Rasperini G (2020). Aesthetic- and patient-related outcomes following root coverage procedures: a systematic review and network meta-analysis. J Clin Periodontol.

[16] Tavelli L, Barootchi S, Stefanini M, Zucchelli G, Giannobile WV, Wang HL (2023). Wound healing dynamics, morbidity, and complications of palatal soft-tissue harvesting. Periodontol 2000.

[17] Miron RJ, Shirakata Y, Ahmad P, Romandini M, Estrin NE, Farshidfar N (2025). 30 years of enamel matrix derivative: mimicking tooth development as a clinical concept. Periodontol 2000.

[18] Miron RJ, Sculean A, Cochran DL, Froum S, Zucchelli G, Nemcovsky C (2016). Twenty years of enamel matrix derivative: the past, the present and the future. J Clin Periodontol.

[19] Shirakata Y, Nakamura T, Shinohara Y, Nakamura-Hasegawa K, Hashiguchi C, Takeuchi N (2019). Split-mouth evaluation of connective tissue graft with or without enamel matrix derivative for the treatment of isolated gingival recession defects in dogs. Clin Oral Investig.

[20] Tonetti MS, Fourmousis I, Suvan J, Cortellini P, Brägger U, Lang NP (2004). Healing, post-operative morbidity and patient perception of outcomes following regenerative therapy of deep intrabony defects. J Clin Periodontol.

[21] Xiang C, Zhang L, Tao E (2025). Research progress of enamel matrix derivative on periodontal tissue regeneration: a narrative review. Front Dent Med.

[22] Paolantonio M (2002). Treatment of gingival recessions by combined periodontal regenerative technique, guided tissue regeneration, and subpedicle connective tissue graft: a comparative clinical study. J Periodontol.

[23] Cetiner D, Parlar A, Balos K, Alpar R (2003). Comparative clinical study of connective tissue graft and two types of bioabsorbable barriers in the treatment of localized gingival recessions. J Periodontol.

[24] Zucchelli G, Clauser C, Sanctis M, Calandriello M (1998). Mucogingival versus guided tissue regeneration procedures in the treatment of deep recession type defects. J Periodontol.

[25] Yarkaç FU, Sen DÖ, Yildirim K, Eroglu ZT, Babayigit O (2025). Comparison of clinical and esthetic results of different techniques in the treatment of multiple gingival recessions. J Esthet Restor Dent.

[26] Silness J, Löe H (1964). Periodontal disease in pregnancy II: correlation between oral hygiene and periodontal condition. Acta Odontol Scand.

[27] Bozkurt Dogan S, Öngöz Dede F, Balli U, Atalay EN, Durmuslar MC (2015). Concentrated growth factor in the treatment of adjacent multiple gingival recessions: a split-mouth randomized clinical trial. J Clin Periodontol.

[28] Din F, Kabalak MÖ, Yilmaz BT, Baris E, Avci H, Çaglayan F (2025). Efficacy of different gingival graft de-epithelialization methods: a parallel-group randomized clinical trial. Clin Oral Investig.

[29] Cairo F, Rotundo R, Miller PD, Pini Prato GP (2009). Root coverage esthetic score: a system to evaluate the esthetic outcome of the treatment of gingival recession through evaluation of clinical cases. J Periodontol.

[30] Aroca S, Zucchelli G, Di Domenico GL, de Sanctis M (2025). Decision tree for the treatment of multiple gingival recession defects when utilizing MCAT or MCAF based on evidence and clinical experience. Int J Periodontics Restorative Dent.

[31] Górski B, Górska R, Wysokinska-Miszczuk J, Kaczynski T (2020). Tunnel technique with enamel matrix derivative in addition to subepithelial connective tissue graft compared with connective tissue graft alone for the treatment of multiple gingival recessions: a randomized clinical trial. Clin Oral Investig.

[32] Mauricio JM, Furquim CP, Bustillos-Torrez W, Soto-Peñaloza D, Peñarrocha-Oltra D, Retamal-Valdes B (2021). Does enamel matrix derivative application provide additional clinical benefits in the treatment of maxillary Miller class I and II gingival recession? A systematic review and meta-analysis. Clin Oral Investig.

[33] Stähli A, Duong HY, Imber JC, Roccuzzo A, Salvi GE, Katsaros C (2023). Recession coverage using the modified coronally advanced tunnel and connective tissue graft with or without enamel matrix derivative: 5-year results of a randomised clinical trial. Clin Oral Investig.

[34] Shanbhag S, Stødle IH, Lie SA, Sanz M, Verket A (2025). Histological outcomes of root coverage procedures: a systematic review with meta-analysis. J Periodontal Res.

[35] Bruno JF, Bowers GM (2000). Histology of a human biopsy section following the placement of a subepithelial connective tissue graft. Int J Periodontics Restorative Dent.

[36] Górski B, Górska R, Szerszen M, Kaczynski T (2022). Modified coronally advanced tunnel technique with enamel matrix derivative in addition to subepithelial connective tissue graft compared with connective tissue graft alone for the treatment of multiple gingival recessions: prognostic parameters for clinical treatment outcomes. Clin Oral Investig.

[37] Imber JC, Kasaj A (2021). Treatment of gingival recession: when and how?. Int Dent J.

[38] Trullenque-Eriksson A, Derks J, Andersson JS (2023). Onset of periodontitis: a registry-based cohort study. Clin Oral Investig.

[39] Stähli A, Imber JC, Raptis E, Salvi GE, Eick S, Sculean A (2020). Effect of enamel matrix derivative on wound healing following gingival recession coverage using the modified coronally advanced tunnel and subepithelial connective tissue graft: a randomised, controlled, clinical study. Clin Oral Investig.

[40] Henriques PS, Pelegrine AA, Nogueira AA, Borghi MM (2010). Application of subepithelial connective tissue graft with or without enamel matrix derivative for root coverage: a split-mouth randomized study. J Oral Sci.

[41] Aroca S, Di Domenico GL, Darnaud C, de Sanctis M (2021). Modified coronally advanced tunnel technique with site-specific application of connective tissue graft for the treatment of multiple adjacent maxillary gingival recessions: a case series. Int J Periodontics Restorative Dent.

[42] Zucchelli G, Tavelli L, McGuire MK, Rasperini G, Feinberg SE, Wang HL (2020). Autogenous soft tissue grafting for periodontal and peri-implant plastic surgical reconstruction. J Periodontol.

[43] Cabrera P, Fernandes GV (2025). A 37-year retrospective assessment of connective tissue grafting: what have we learned? A case report. Int J Periodontics Restorative Dent.

[44] Stefanini M, Zucchelli G, Marzadori M, de Sanctis M (2018). Coronally advanced flap with site-specific application of connective tissue graft for the treatment of multiple adjacent gingival recessions: a 3-year follow-up case series. Int J Periodontics Restorative Dent.

[45] Lee JH, Kim YT (2025). Modified tunnel technique with and without enamel matrix derivative for deep and narrow gingival recession in the mandibular anterior region: a 3-year longitudinal and retrospective cohort population-based study. J Periodontal Implant Sci.

[46] Bakhishov H, Isler SC, Bozyel B, Yildirim B, Tekindal MA, Ozdemir B (2021). De-epithelialized gingival graft versus subepithelial connective tissue graft in the treatment of multiple adjacent gingival recessions using the tunnel technique: 1-year results of a randomized clinical trial. J Clin Periodontol.

[47] Tavelli L, Barootchi S, Cairo F, Rasperini G, Shedden K, Wang HL (2019). The effect of time on root coverage outcomes: a network meta-analysis. J Dent Res.

[48] Elbana A, Saleh W, Youssef J (2025). Comparing connective tissue grafts and collagen matrix in modified coronally advanced tunnel technique for RT1 gingival recession: a randomized controlled clinical trial. BMC Oral Health.

[49] Würflein E, Ollinger S, Sculean A, Vach K, Landwehr VC, Nelson K (2025). Modified coronally advanced tunnel technique with porcine dermal matrix for recession treatment: 12-month follow-up. Clin Exp Dent Res.

[50] Mayta-Tovalino F, Barboza JJ, Pasupuleti V, Hernandez AV (2023). Efficacy of tunnel technique (TUN) versus coronally advanced flap (CAF) in the management of multiple gingival recession defects: a meta-analysis. Int J Dent.

